# A Review on Sustainable Recycling of NdFeB Waste: Methodologies, Challenges, and the Integration of Machine Learning (ML)

**DOI:** 10.3390/ma19030594

**Published:** 2026-02-03

**Authors:** Rehan Ullah, Jason Daza, Asma Wederni, Lluisa Escoda, Joan Saurina, Joan-Josep Suñol

**Affiliations:** Department of Physics, Universitat de Girona, P2, EPS, Campus Montilivi s/n, 17003 Girona, Spain; rehan.ullah@udg.edu (R.U.); jason.dazac@udg.edu (J.D.); asma.wederni@udg.edu (A.W.); lluisa.escoda@udg.edu (L.E.); joan.saurina@udg.edu (J.S.)

**Keywords:** rare earth elements (REEs), neodymium iron boron (NdFeB) waste, recycling process, pyrometallurgy, hydrometallurgy, machine learning (ML), sustainability

## Abstract

The increasing demand and production of neodymium-iron-boron-based permanent magnets (NdFeB-PMs) for the electronics, energy sector, and automobile industries led to disposal consequences. The NdFeB-PMs contain a substantial amount of rare earth elements (REEs). Although China is the largest exporter of REEs to the world, it has applied some restrictive policies in terms of supply chain and taxes. To address such issues, this review systematically examines current recycling techniques, including short-loop, hydrometallurgy, pyrometallurgy, and hybrid processes, and the integration of Machine Learning (ML) to the leaching process, with a particular focus on their impact on industrial capability, economic viability, and environmental concerns. However, a comparative study highlights ongoing challenges to large-scale implementation, including fragmented waste sources, gaps between efficient processes and environmental sustainability, and a lack of regulatory and infrastructure support. To address these challenges, technical innovation in automated disassembly systems and selective REE recovery via ML was discussed, along with legislative initiatives such as Extended Producer Responsibility (EPR) and waste monitoring procedures. Furthermore, ecologically and economically feasible solutions were optimized through ML-based recycling procedures to increase the leaching efficiency and the recovery of the REEs. This analysis emphasizes the importance of collective technological, environmental, and policy initiatives to achieve sustainable NdFeB recycling and long-term resource availability. These findings offer important perspectives into developing effective and environmentally friendly NdFeB waste recycling solutions via the integration of ML.

## 1. Introduction

**Figure 1:**
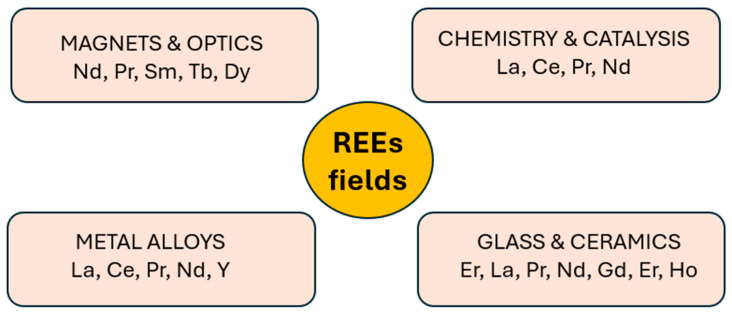
REEs are traditional fields and elements for many modern technologies.

A worldwide consensus has grown on sustainability, which includes solar cells, wind energy, electric vehicles (EVs), and energy storage devices, in response to the intensifying political and public pressure to tackle climate change [1]. For instance, the International Energy Agency (IEA) estimates that the proportion of EVs in worldwide automobile sales is increasing, as measured from 5% to 60% by 2030 [2]. In addition, solar and wind power plants are expected to account for 70% of the world’s electricity production by 2050, contributing to the predicted 90% renewable energy production [3]. The demand for critical metals, such as rare earth elements (REEs), has significantly increased by the worldwide shift towards environmentally friendly and renewable energy sources [4]. REEs are the fundamental components of renewable energy systems, as they are utilized in EV motors and magnets in wind turbines [5]. Figure 1 describes the main applications of REEs.

The REEs are known as the industrial vitamins because of their distinctive magnetic, electrochemical, and optical properties [6]. There is a group of 17 elements of REEs, such as scandium, yttrium, and the other 15 lanthanides, which have similar physical and chemical characteristics. The European Union (EU) has classified these elements as critical raw materials as a result of their economic significance and supply risk, which are influenced by factors such as the dominance of production in regions with poor governance, limited substitution capability, and lower end-of-life recycling capacities [7]. These elements are widely used in various sectors, including automobile manufacturing, electronic devices, fuel cells, residential appliances, petrochemical technology, and defense applications [8]. In electronics and renewable energy systems, electric motors and generators are essential parts with exchangeable energy conversion systems. The substantial assembled permanent magnets (PMs) utilized in EVs’ motors and generators are manufactured of REEs such as Neodymium (Nd), Dysprosium (Dy), Praseodymium (Pr), and Terbium (Tb) [9].

The REEs are split into three main categories: heavy, medium, and light. Heavy REEs (H-REEs) are more economically significant than other REE categories due to their diverse applications. Likewise, H-REEs are often less frequent in REE minerals than L-REEs and M-REEs, making them very rare in terms of relatively high occurrence. Therefore, it is important to separate and collect H-REEs from both L-REEs and M-REEs by maintaining their significant economic value and confirming market demand. Given the overall scarcity and need for REE materials, it is necessary to recover all three types of REEs [10]. Importantly, the L-REE/H-REE classification does not apply to scandium, a radioactive assigned light lanthanide, and the unique promethium (P_m_) [11]. The P_m_ is exceptional among these REEs as it rarely occurs in nature [12]. The deposits, mainly peralkaline, alkaline, and carbonatite, are found in REEs reserves. The extraction of these elements is extremely challenging, often resulting in large amounts of hazardous and highly toxic waste. Additionally, regional tax policies, stockpile strategies, consolidation of industries, and production regulations can have a substantial impact on the market prices of REEs and worldwide supply chains [13].

**Figure 2:**
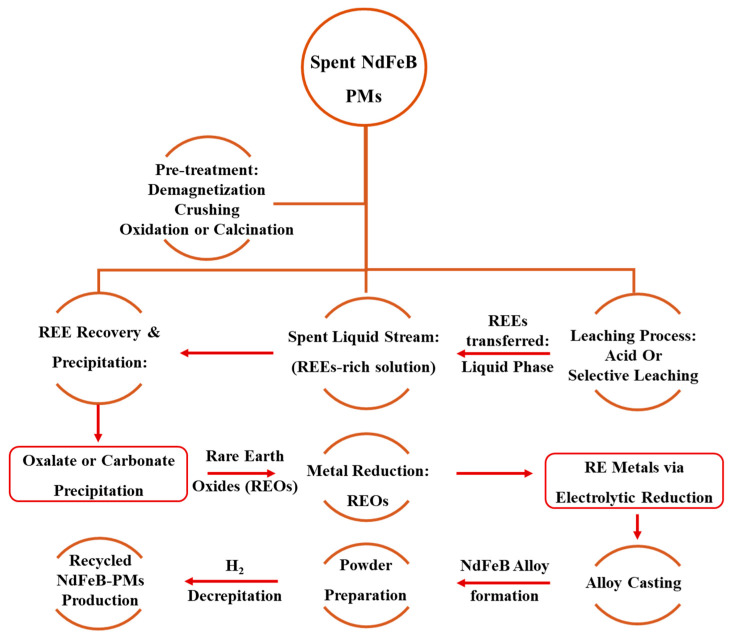
Schematic flowchart of the industrial recycling steps for NdFeB magnetic waste to the re-manufacturing of magnets.

Recycling PMs containing REEs on an industrial scale is a relatively new concept globally. For example, the spent liquid stream, which contains the REEs, is reduced to Rare Earth Oxides (REOs). Subsequently, additional steps for REOs, such as metal reduction, alloy casting, and powder preparation, need to be employed for magnet production as described in Figure 2. However, other companies, focusing on technical advancements and industrialization, have also grown. The majority of Chinese businesses mostly use the dismantling, de-magnetization, disintegration, and crushing (D^3^C) technique for the separation of PMs into their single elements. On the other hand, American and European industries tend to prioritize recycling technologies by making it a more direct pathway. The HPMW, known as Hydrogen Processing Magnetic Waste, is used to recycle PMs, which are subsequently employed for the sintering and magnets bonding. It is announced that a German plant will be opened soon, which is expected to have a recycling facility of around 100 to 330 tons annually. Similarly, a US-based magnetic company (Noveon Magnetics, San Marcos, Texas, USA) with a yearly capacity of 2000 tons, uses HPMW in conjunction with grain-boundary engineering solutions. Meanwhile, the French Carester company (Lyon, France) is in the process of advancing hydrometallurgical separation technology. The management of magnetic wastes around 2000 metric tons per year by 2027, it intends to construct a pilot plant by 2026 [14]. Thus, electrification is more than a technological upgrade; it lowers combustion and overall greenhouse gas (GHG) emissions, decreasing atmospheric GHG accumulation and global warming. In general, REEs are essential non-renewable vital resources that significantly impact the sustainable development of modern civilization, sciences, and technology [15]. Therefore, an integrated approach is needed to ensure the long-term viability of the REEs industries, which includes not just technological aspects like extraction technology and production efficiency, but also non-technical concerns like economic feasibility, geopolitical viability, and regulatory structures.

The present review carefully describes the present and forthcoming perspectives of sustainable development for the recovery of RE alloys, metals, and compounds from NdFeB magnetic waste. Nevertheless, there is a significant absence of comprehensive research in industrial recycling technologies (e.g., Hydrometallurgy, Pyrometallurgy, and short loop) and the ecological aspects that emphasize the systematic evaluation of integrated parameters in these emerging techniques. Initially, in the literature, the significant research gaps were addressed by highlighting the metal recycling methods individually and their recovery efficiencies, following their industrial implications and environmental goodness. In addition, the sustainable and effective solutions for recovering REEs were addressed through the implementation of the pretreatment of recycling for magnetic wastes and modifying the leaching parameters via an ML-based Graphical User Interface (GUI) model. Likewise, the optimized parameters for the effective leaching, based on particle size, waste composition, and calcination parameters in the hydrometallurgical procedure, were analyzed via Partial Dependence Plot (PDP) and Shapley Additive Explanations (SHAP), and highlighted their environmental effectiveness.

## 2. The Future Economic Importance of REEs

The European Union’s (EU) plan for 2050 to become a competitive, circular, and climate-neutral economy is mainly based on the EU’s capacities for the execution of sustainability and EVs initiatives in an environmentally friendly and economically feasible approach. All equipment, whether commercial or residential, will need to meet the rigorous protocols for energy efficiency. The EU research report indicated that, by 2023, the annual demand for REEs from electric vehicles alone was approximately 1.5 × 10^7^ kg, corresponding to 1 kg of REE per vehicle. The REEs typical elemental composition (wt.%) is used for the production of permanent magnets, as shown in Table 1, where the terbium (Tb) and other REEs are typically present in small amounts depending on the magnet’s grade and application. Although REEs are used in many applications, only 25% of the total production of these chemical elements is used in the production of magnetic material. However, they dominate the market value, representing approximately 80–90% of the global REE market [16].

The relatively small market of RE-PMs, around €6.5 billion, still has a significant influence. It is estimated that the EV industry (i.e., EU27) is expected to rise to around €500 billion by 2030, resulting in the support of 6 million jobs [18]. The Joint Research Center of the EU has transformed the climate goals for 2030 and 2050 into yearly raw material demands through modeling. The demand for REEs in sustainability and highly innovative industries, which play an important role, is expected to rise by more than 7.5%/year worldwide [19]. The total REEs reservoir globally is around 110 million tons, and is located in China, Vietnam, and Brazil, with their respective volumes of 44, 22, and 21 million tons. A comparison with the present supply for the entire EU economy reveals that by 2030, the three vital sectors of renewable energies, EVs, warfare, and the aerospace industry will require up to 5 times the amount of Dy and approximately 4 times the amount of Nd. By 2050, the demand might be expected to increase to 13 times the current supply [7,20].

**Figure 3:**
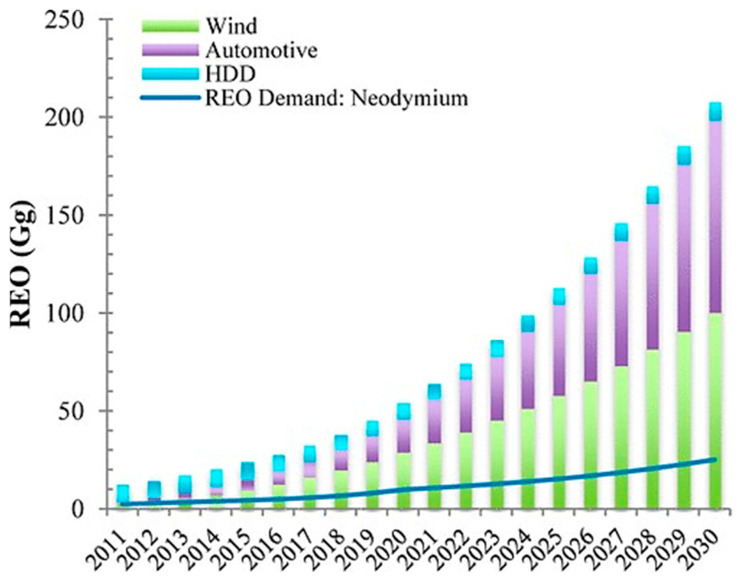
Demands for the potential value in Gg of REO for magnets for advanced technologies, reprinted from [23].

The EU is considered the essential competitor of the production of PMs, whose imports mainly rely on the entire value chain of REEs-based magnets. The rare-earth oxides (REO) demand is steadily increasing for magnet applications, as shown in Figure 3. The constant demand and upward trend indicate a further increase in EOL flows after 2030. However, in 2021, China manufactured around 93% of the 0.14 million tons of RE-PMs produced globally, indicating a highly dominant production. This dominance of the RE worth chain, all stages from mining to recycling, is observed thoroughly. In contrast to the 16,000 tons of magnets imported annually from China, Europe’s remaining production capacity of 1000 tons is struggling to keep up. The effective recycling of Nd, Dy, Pr, and Tb in NdFeB magnets, with the potential to exceed known geological reserves by nearly 3 times when benchmarked against the annual extraction rates reported in 2007 [21]. These reserves for each element are 62.6 (Nd), 15.7 (Dy/Pr), and 3.1 (Tb) kilotons, respectively. The capacity of European magnet production is underutilized and is primarily used for specialized, particular applications, despite the expanding market. There has been an increase in the import of magnets containing REEs for usage in motor and generator components and appliances. EVs are primarily composed of metallic alloys, unlike Li-ion batteries, which can be reprocessed and recycled through conventional approaches. However, the main obstacle is disassembling both the metallic alloys and batteries to obtain valuable metals, but there is the possibility of destroying them when using less selective recycling processes [22]. So, to tackle these challenges encountered by the electrification sector, it is necessary to study possible EM topologies and alternative magnetic materials to the hazardous, cost-volatile, and ecologically destructive sintered NdFeB magnets.

## 3. The Recycling Sources of REES from Waste

**Figure 4:**
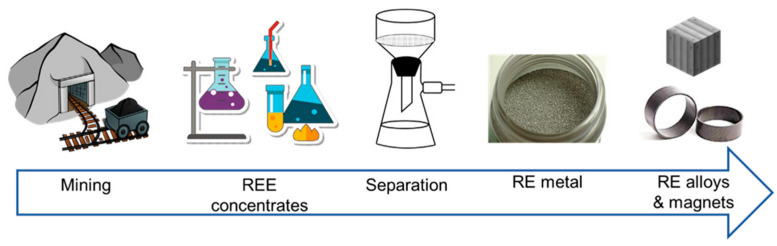
Steps in the processing and refinement of REEs [16].

The REEs are obtained from mining. Figure 4 shows the steps in the processing and refinement of REEs [16].

The REEs’ demands in renewable energy and other technologies are expected to grow at a rate exceeding 7.8 to 8.5% annually [24]. Thus, recycling is an increasing field of research. In 2024, the Department of Energy (DOE) classified some REEs as critical materials. The compressor motors from cooling devices, robotics, and separators are additional likely sources of residue NdFeB PMs. The EVs’ motors are also a good source of recycling metals, including Fe, Cu, and REEs like Nd and Dy. The estimated number of EVs in existence is 14 million units as of 2021 [25], and by 2033, it is predicted to reach a remarkable 15 million units per year [26]. The REEs in one EV contain Nd, Dy, and Pr mixture elements by estimating up to 0.6–2 kg or (27–32 wt.%) in the NdFeB PM [27], indicating these constituents are the main target materials for extraction and recycling. In 2023, the EV sales were close to 14 million, up 35% from the previous year and over 6 times greater than in 2018. To recycle REEs from the EV motor magnets, Nissan Motor Corporation started using pyrometallurgy [28]. According to Ghorbani et al. [29], more than 85% of the market is accounted for by both China and Europe combined. Since 2010, NdFeB consumption in consumer electronics has decreased due to the increase in solid-state drives (SSDs), even though mechanical HDD use has been growing due to increasing data storage demands. Nonetheless, HDDs have a short-term recycling rate, with a 50% Nd extraction ability in the initial production [17]. In comparison with novel NdFeB applications, their standard features and existing supply networks make REE recovery more efficient.

The majority of the Life Cycle Assessment (LCA) analyses on PM recycling have utilized EOL magnets extracted from Hard Disk Drives (HDDs), with a small number also utilizing magnets from EV motors [30]. Zhao et al. [31] found that by 2030, EOL trash will have surpassed production-derived waste to rise to the most widely used secondary resource. The recycling of NdFeB PMs globally might reach 18–22% of Nd and Pr demands and 20–23% of Dy and Tb needs for NdFeB manufacturing. The NdFeB magnet recycling assumptions range from 3 to 178 kg CO_2_/kg, suggesting the need for standardized methodologies and a large data gap. There has been a total of 450 × 10^3^ and 11 × 10^3^ kg of Nd and Dy recycled in 2020. The NdFeB magnets are presently utilized in electric motorcycles (EMCs), which constitute approximately 8% of the global NdFeB demand. The usage of REEs is massive due to the 400 million EMCs in metropolitan China’s inventory by the year 2026. The EMCs’ NdFeB magnet usage will increase due to short lifespans and rising consumer expectations.

The most widely used PMs are NdFeB due to its unique characteristics compared to Ferrite, AlNiCo, and SmCo, as discussed in the comparative Table 2. In particular, the market share of PMs of NdFeB (48–64%), ferrite (28–32%), SmCo (5–7%), and AlNiCo type (2–6%) is approximately [32]. Different industries have different perspectives on the recycling possibilities of EOLs’ waste. Although there will be no instant recycling opportunities and huge long-term material stockpiles due to the widespread utilization of PMs (i.e., 1–2 tons per megawatt), the expansion capacity will increase in three decades. According to the International Wind Energy Council, it is expected that installed windmill capacity might increase from 743 to 1120 GW by 2025. The amount of NdFeB magnets needed for each turbine is around 232 kg [33]. Schoonfeldt et al. [34] predict that wind turbine generators will be more commonly recycled commencing about 2030.

## 4. The Market Demand for NdFeB and SmCo Recycled Magnets

The three primary areas addressed by the literature are magnets’ physicochemical qualities, recycling methods, and the effects on the environment and economic growth. Therefore, the following sections are the main points of this article: the nature of NdFeB waste, the effects on the environment and the economy, and a comparison of recycling processes. The majority of emerging approaches were assessed and discussed from the latest literature [17] that shows aptitude for sustainability and recovery effectiveness.

There are various techniques to categorize recyclable NdFeB materials. The REEs categories were identified on the basis of REEs elements, which were 30 wt.% of RE in low RE PMs waste [35]. Additionally, the NdFeB waste was classified based on its resources into two groups: One group is the waste collected from production. This group includes sintering and milling of materials, chopping the scrap and sludge from PMs, cutting, machining, and shaping [36]. The nonuniform distribution of particles from these wastes ranges from 0.1 to 5 mm in size, and quite homogeneous composition (Nd amount: 15–35 wt.%) due to minimal ambient exposure. The Sludge, on the other hand, requires pre-treatment, such as cleaning or thermal processing, due to its high oxidation level and the abundance of solid and liquid contaminants [37]. Likewise, the second one is waste from end-of-life (EOL) products. It is manufactured from parts that have been rendered obsolete, such as HDDs, household electronics, and magnetic motors. The recycling accessibility of these vital minerals is limited, but future forecasts highlight NdFeB magnets as crucial secondary RE sources.

In particular, SmCo recycling encounters an additional challenge: its production capacity is comparably low. Magnets made of SmCo_5_ or Sm_2_Co_17_ alloys account for just 2% (2 or 3 kilotons) of the total supply expected in a year [38]. Likewise, the EOL magnetic waste is mainly composed of NdFeB alloys, with a small amount of SmCo magnets, and does not contain RE-based magnets such as BaFe_12_O_19_ and ferrites. Additionally, each magnet has encountered distinct remanence losses during its specific application, which impedes the process of effectively separating these magnets by arranging them based on their residual density of magnetic flux [39]. To sum up, there is currently no method to recover Sm and Co from large amounts of pre-separated SmCo materials. Therefore, recycling NdFeB magnets is the easiest way to reach cost-effective SmCo recycling. Table 3 shows the global market share (in wt.%) of main REEs in NdFeB and SmCo PMs.

## 5. The NdFeB Magnets’ Properties

The NdFeB PMs are mainly composed of Nd and Fe with small quantities of other REE elements, like Sm, Pr, and La. The optimum performance of magnets for specific applications can be achieved by adding various elements. As an example, Dy and Tb improve the magnet’s performance at high temperatures by enhancing coercivity and anisotropy. As Cobalt (Co) increases in the Curie temperature (T_c_), the inherent coercivity and thermal processing performance are enhanced by gallium (Ga). Generally, the microstructure is refined, and performance is improved by adding Boron (B) approximately 1 wt.%. To analyze the structure of NdFeB magnets, which is primarily composed of three major discrete phases: The Nd, B, and NdFeB matrix phases. The Nd_2_Fe_14_B matrix phase, having tetragonal crystallites of the P42/mnm space group, is the hardest magnetic part of the Nd-rich phase, which is composed of approximately 80% of Nd, Nd_4_Fe, and Nd_2_O_3_, and exists mainly at the grain limits of the primary phase. The Nd-rich phase, which is a non-magnetic dopant, increases the magnet’s intrinsic coercive force and its density [40]. The Nd-rich phase consists of the Pr, which helps the equal dispersion of the rich Nd- phase and impedes the magnet’s disintegration [41]. During production, a B-rich phase is typically formed when the magnet’s B content rises beyond 7%. The B-rich phase is observed as large particles at the grain boundaries, while it is present as fine particles precipitating within the grains [42]. The Nd-rich phases have higher chemical reactivity and lower corrosion resistance, having intergranular spaces located at the grain boundary and triple junction that are mostly exposed to chemical reagents. Consequently, the chemical solubility reaction starts at the intergranular spaces, which dissolve the rich phases of Nd more efficiently [43]. Thus, leaching starts at the grain boundaries before penetration to grains enables RE extraction and reaction kinetics through their high surface energy and defect density at grain boundaries.

## 6. The PMs-NdFeB Waste Recycling Technology Challenges

The NdFeB magnets’ recyclability, scrap, or trash is extremely material-dependent. Currently, the most environmentally and economically beneficial method for NdFeB waste is direct recycling in their degraded condition, as they are scarcely contaminated, oxidized, or corroded.

The re-sintering [44] and the HDDR, known as the Hydrogenation Disproportionation Desorption and Re-combination [45], are two examples of magnet-to-magnet procedures that produce new magnets that can be reused in prior systems or applications. The total volume of the non-ferromagnetic phases, such as RE oxides, increased with each recycling cycle of NdFeB magnet wastes because they are not separated during recycling. Consequently, NdFeB magnets produced through these recycling procedures often have worse magnetic characteristics depending on how many cycles they perform [46]. As a result, new raw materials are still preferred by the magnet sector for production to guarantee the desired magnetic strength.

## 7. Pre-Recovery Processing of Waste

The REEs’ efficient extraction from NdFeB PMs, especially those contained in the intricated EOL products, requires physical separation technologies. These products are typically subjected to disintegration after the elimination of toxic ingredients. Additionally, REE recovery becomes economically impractical when tiny magnets are attached to the ferrous metal due to their enormous magnetism [47]. The different shredding techniques have different effects on magnet recovery productivity. A magnet concentration recovery rate of around 70% can be achieved through precise shredding, where the small particles obtained from splitting the magnet can be collected efficiently using a 30 mm steel grate. This compares favorably with coarse shredding, which can result in weakly freed magnets [48].

There have been new approaches to pre-processing e-waste in an effort to increase the recovery of REEs. The automated process of the recovery of REEs from HDDs, which includes the disassembling and recycling of permanent magnets, is the main objective of the European VALOMAG project [49]. Similarly, the REEs from magnets through direct recycling have been made possible by the development of automated systems that disassemble the e-waste and are investigated at a US laboratory named Oak Ridge National Laboratory (ORNL) [50]. The challenges of magnet recovery before shredding have prompted the development of various approaches [51]:

Manual Disassembly: This method is a labor-intensive separation approach in which magnets are physically removed from EOL products by using semi-machined tools for assistance. Additionally, this method is primarily feasible for the large-scale components such as NdFeB magnets used in wind turbines, traction motors for EVs, and industrial machinery. In such cases, manual disassembly allows selective recovery of magnets with minimal contamination, preserving their magnetic and structural integrity for direct reuse and processing.

Mechanical Separation: This approach has emerged as an efficient and scalable approach for recovering PMs from EOL products. Companies such as Mitsubishi and Hitachi have developed advanced mechanical separation systems integrated with machine-learning techniques for the selective recovery of magnets from ACs and HDDs. In addition, these systems are designed to identify the rich magnet components and optimize the disassembly pathways without damaging the structure of magnets. These techniques avoid damaging the magnets while extracting screws and separating them from their cases by using vibration, impact, and rotating drums [52].

Magnetic Waste via Hydrogen Processing (HPMS): This process involves H_2_ reaction with sintered NdFeB magnets by creating a hybrid phase at the grain boundary. It causes the decomposition of magnets into powders that can be instantly extracted from other constituents [53].

The Advancing and Pre-disassembling of HDDs: Certain companies prioritize the disintegration of pre-disassembled HDDs in order to improve data security by concentrating on the magnets. The utilization of a magnetic grid system in conjunction with a fine shredder has yielded excellent results in the separation and concentration of magnet particles from disintegrated HDD components [48]. Multiple factors determine whether magnets can be separated before shredding. Initially, removing magnets is crucial, necessitating an efficient removal of adhesives, fasteners, and other parts that anchor the magnets inside the apparatus. The demagnetization procedure is essential for the subsequent treatment process [54].

## 8. The NdFeB Magnetic Waste Recycling Technologies

**Figure 5:**
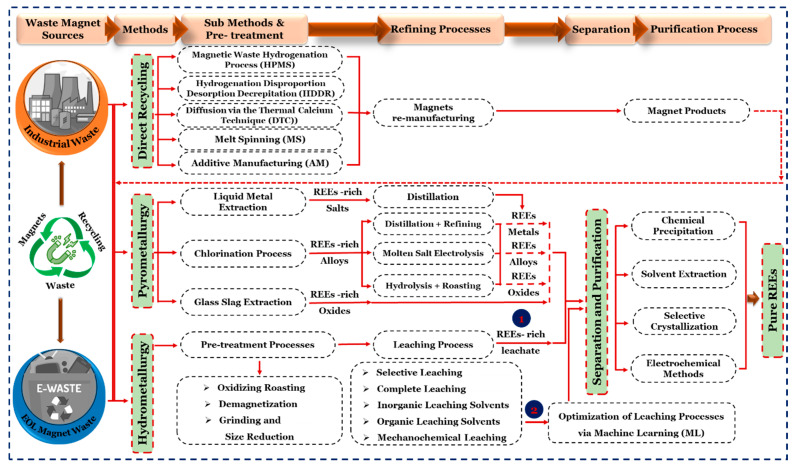
The complete summary of the recycling techniques, followed by pre-treatments and purification steps. 1 and 2 remark the two main pathways of the hydrometallurgy route.

The recycling of NdFeB PMs involves three main methods for recycling PMs: Hydrometallurgy, Pyrometallurgy, or direct recycling using hydrogen (H) decomposition, as shown in Figure 5. The extraction of REEs from NdFeB waste is made more difficult by a variety of factors, including heterogeneity, oxidation, brittleness, intense magnetic forces, and the acquisition of appropriate samples in NdFeB magnet recycling. Some methods for recycling NdFeB involve metal-to-metal (MtM), waste-to-alloy, waste-to-REE, and direct recovery. The NdFeB magnets were initially broken down and shredded until they reached 30 mm in size. The productivity of recycling is considerably impacted by labor-intensive physical disassembly, demagnetization, chemical detachment, thermal breakdown of adhesive connections, and handling technologies [55]. The demagnetization procedure begins with heating the material to 300 to 500 °C in an air or Ar environment before size reduction. The subsequent procedures are followed as grinding, screening, hydro/pyro, or electrometallurgy separation. In comparison to the Virgin manufacture (primary production of NdFeB magnets from raw RE-ores), the metal-to-metal (MtM) route involves limited process phases.

### 8.1. Hydrogenation-Induced Decomposition of NdFeB Magnets in Recycling Processes

The hydrogenation process mainly starts at the triple junctions of the Nd-rich grain boundary phases in NdFeB waste, where atomic disorder, high free volume, and chemical reactivity facilitate the hydrogenation [56]. In addition, as the hydrogen is added, the HPMS procedures develop the intrinsic micro-structural contrast between the Nd-rich intergranular phases and NdFeB grains, which display noticeably different thermodynamic and hydrogen absorption kinetics. Similarly, the Nd-rich phases instantly form Nd-hydrides, enduring a significant expansion in volume, while the NdFeB grain absorbs a lower content of H_2_. Subsequently, the localized expansion develops internal stresses at the grain boundaries and triple junctions, resulting in intergranular de-cohesion and microcrack formation. Thus, the overall conversion from bulk magnets into powder is facilitated by the preferential H_2_-assisted expansion of Nd-rich phases, paired with the nucleation and growth of the Nd-H hybrid phases, leading to the propagation of cracks throughout the micro-structure [57]. It is imperative to discuss the two existing strategies of Hydrogenation methods, HD and HDDR, separately, because of their similar terminology and some variations.

### 8.2. The Magnetic Waste Hydrogenation Process (HPMS)

The NdFeB recycling of EOLs (primarily HDDs) is the subject of technical and technological developments, with a focus on hydrogenation-based MtM recycling. This approach has many benefits, such as lowering supply uncertainty and reducing energy usage. In comparison with conventional magnet manufacturing from ores, this strategy uses 45% less energy, which makes it more affordable (production costs are reduced by 53%) and ecologically sustainable (CO_2_ emissions are reduced by more than 11 tons/ton of recycled magnets) [58]. It is feasible to recycle HDD magnets from waste parts in two steps: initially, by collecting and grinding the EOL magnets into powder, and then, process the fresh NdFeB PMs.

Initially, the magnetic parts from waste are disassembled via HDDs either manually or auto machine to separate the scrap. Consequently, they developed the demagnetization of the assembled unit before wrapping it up for the hydrogenation process. Consequently, the homogenization of NdFeB EOL particles is produced by around 5% after the hydrogen blending of REEs. Likewise, the mixture of particles was again milled and homogenized. Finally, the obtained products of slabs undergo magnetization and are sintered [59]. The new slabs’ efficiency and magnetic properties exceed 90% as compared to those manufactured from ores [60].

The HPMS has been adopted over the years as it is one of the most effective procedures for the recycling of MtM. Nevertheless, it is unable to gain the interest of recycling companies and replace the existing separation processes. The production of NdFeB PMs involves hydrogen decrepitation (HD), which is the initial stage before grinding, which is used for the conversion of alloys into particles [61]. To receive the uniform and ultrafine grains, the second method is HDDR, as described and discussed below.

### 8.3. Hydrogen Decrepitation and De-Hydrogenation Techniques

The penetration of a reactive hydrogen atom is only possible for different. metal crystalline grain boundaries. The prevention of fracture and brittleness in the typical presence of metallurgy, the hydrogenation is prevented from permeating the metal. Hydrogen can swiftly diffuse into grain boundaries, exerting stress at the weakest region, thereby putting in motion micro-cracks that progress through the grain structure. The propagation enables the specimen to encounter inelastic strain by considerably reducing the fracture strength [62]. The targeted hydrogenation technique decomposes and degrades several materials using hydrogen’s capability.

The NdFeB PMs recycling and manufacturing are facilitated by the use of hydrogen decrepitation (HD) at ambient and lower temperatures. Consequently, the disintegration of the total microstructure exclusively in powder form leads to a reduction in particle size. The HD mechanism operates at a temperature of 25–400 °C, which leads to small particles ranging from 6 to 600 μm by following hydrogenation [63]. In comparison to other methods, including mechanical crushing, the major benefit of the HD process is that it produces a hydrogenated powder that is both exceptionally flexible and demagnetized before jet milling [64].

### 8.4. The Diffusion via the Thermal Calcium Technique (DTC)

The DTC approach for the objectives of NdFeB recycling through sintering attracted substantial attention, as it provides specific benefits in terms of ecological sustainability, energy preservation, and process effectiveness. Yue et al. [65] observed that the scarped sludge passes from the intense oxidation process and is polluted with oil, water, and solid matter produced by the equipment. As a result, it cannot be directly used to fabricate NdFeB sintered magnets. Consequently, the byproduct experiences initial purification through the use of distillation and washing procedures. Further, the DTC technique is used to create NdFeB particles with a single crystal. To eliminate calcium (Ca), H_2_O, methanol, or NH_4_Cl, or acetylacetone is applied to produce the NdFeB PMs by applying traditional sintering procedures.

The traditional DTC process, which utilizes metallic calcium as a reductant, compels the continuous operation at high temperatures (1000–1100 °C), which offers a dual challenge of high chemical costs and substantial energy consumption. The Calcium hydride (CaH_2_) has been considered as a possible reduction agent by recent research. By using CaH_2_, which exhibits a low melting point, superior thermodynamic characteristics, and the ability to release hydrogen, NdFeB powder formed with smaller particles and better dispersion. There is also a considerable decrease in the presence of contaminants, especially the remaining calcium and oxygen concentrations [66].

### 8.5. Melt Spinning (MS)

One efficient method for making magnetic materials from recycled magnets is melting via a spinning process. The procedure involves melting scrap metal via induction melting and then spraying it onto a Cu-wheel that is spinning at a high speed, usually between 15 and 40 m/s. According to Nguyen et al. [67], such methodology leads to incredible cooling rates, around 10^5^–10^6^ K/s, which facilitate the production of amorphous ribbons and nano-crystallinity (5–50 nm). The extremely fast solidification prevents elemental separation and oxidation erosion while keeping the alloy’s desired materials.

To synthesize isotropic NdFeB ribbons, Itoh et al. [68] chose the MS approach and obtained the magnetic performance of the remanence (B_r_ = 0.69 T), coercivity (H_c_ = 0.7 MA/m), and maximum energy (BH)_max_ = 71 kJ/m^3^. In addition, through the MS approach, it is possible to extract magnetic material effectively from sintered NdFeB magnet waste that possesses a coating with Ni. While incorporating Ni instead of Fe decreased the magnetic properties. However, the bonded NdFeB magnets manufactured from the recycled materials were magnetically as effective as commercially available PMs. The anisotropic magnetic materials with grain sizes around 300 nm can be made subsequently by crushing or deforming the material under high temperatures [69]. In conclusion, the NdFeB waste can be reused via this facile and cost-effective approach. The swift cooling inherent to melting the spin enables the development of a nanocrystalline structure, which considerably boosts the magnetic efficiency of recyclable materials.

### 8.6. Additive Manufacturing (AM)

Additive processing of PMs is a cutting-edge innovation that has drawn the interest of both researchers and companies with the goal of lowering the material expenses and expensive manufacturing techniques [70]. Gandha et al. [71] developed a composite powder of NdFeB material and a polymer binder at low temperatures by grinding the particles and crushing the AM waste of PMs. The Recycled magnets produced through hot pressing composite powder exceeded their magnetic behavior in terms of density and magnetic quality. Additionally, the magnetization strength of the magnets was 6.5% higher than the waste magnetization strength of 4%.

## 9. Pyrometallurgical Process

Pyrometallurgical methods include thermal pre-treatment and extraction techniques. These approaches are an appropriate substitute for traditional hydrometallurgical methods of recovering REEs from solid waste. Ramanayaka et al. [72] demonstrate that pyrometallurgy is the most common industrial approach for recovering metal from abandoned electrical and electronic devices. The literature is abundant with reviews of several pyrometallurgical methods for recycling spent NdFeB magnets, including the selective chlorination process, Liquid Metal Extraction (LME), slag refinement, and the Glass-Slag Process (GSP). The direct melting or LME of REE alloys can be conducted without transforming them into oxides by applying certain pyrometallurgical methods. It overcomes problems such as wastewater production and unnecessary usage of chemicals. The processing temperature optimization for the efficient extraction of REEs is very important for electronics components. Thus, thermal pre-treatment generally involves combustion and pyrolysis as initial procedures, whereas extractive pyrometallurgical methods cover processes such as metal melting and roasting.

### 9.1. Liquid Metal Extraction (LME)

In LME, REEs are dissolved selectively in a liquid alloy system, which leads to the formation of two incompatible liquid metal phases. This approach enables the production of intermetallic compounds with a low melting point by taking advantage of the great affinity of specific metals for REEs [73]. The basics of LME are fundamentally similar to the traditional approach of liquid–liquid extraction [74]. In order to remove the REEs from the Fe matrix, these molten metals are used as extracting agents, separating the REEs from scrap NdFeB magnets. An efficient agent for extraction is a metal that can produce an alloy with REEs that has a low melting point, which is insoluble in iron. For example, the research by Tomohiko Akahori et al. [75] utilizes the melted magnesium–aluminum (Mg-Al) as an extracting agent. Whereas, under the optimized conditions at 1000 °C for 6 h with a Mg: magnetic mass ratio of 10:1, selective REEs recovery was achieved, yielding calcium at a Mg: Ca mass ratio of 20:1. The process exhibited near quantitative extraction of Nd (~100%), whereas Dy showed comparatively lower extraction efficiency. The effect on the extracted NdFeB magnetic particles’ size was studied by Na et al. [76] by using molten Mg as the extracting medium. In addition, the utilized magnets were heated to 1000 °C and then mixed with molten Mg for 10–50 min. The solubility of Nd in the Mg melting was observed as the reduction in particle size of waste magnets decreased. The Nd extraction of around 96.5% achieved with a 5 mm particle size and optimum conditions.

Similarly, Sun et al. [77] extracted the Nd from the NdFeB magnet in a pure melted Mg by using the Mg-Nd binary alloys to facilitate the diffusion and produce the reduction process for 30 min. Over the interval of two restoration cycles, an aggregate yield of 80% for Nd extraction was obtained. Additionally, the niobium (Nb) metal was directly extracted from NdFeB magnet waste using molten silver (Ag) by Takeda et al. [78]. Although an Ag-Nd alloy with 40–50 wt.% Nd was formed when the magnets and melted Ag reacted at 1000 °C to extract over 90% of Nd. The Ag-Nd alloy was air-oxidized to convert Nd to Nd_2_O_3_ and separate it from Ag. Meanwhile, the poor sticking behaviors of Nd_2_O_3_ with molten Ag caused total phase separation challenges.

In conclusion, the Mg extraction efficiency for Nd is approximately 100%, whereas that for Dy is below 60% due to Dy’s high oxidative nature and significant metallurgical interaction with the Fe-matrix. The Ag has low process consumption but high cost of materials, limiting its economic viability. While the extractants with low melting points, including bismuth (Bi) and lead (Pb), are likely to be the focus of future research [79]. The Bi is the sole extractant that can recover Nd and Dy from magnet waste at high rates [80].

### 9.2. Chlorination Method

This approach leverages the variant magnetic properties of halide chlorine ions and the unique characteristics of the resulting chlorides to facilitate the removal of REEs and Fe from NdFeB PMs, ultimately forming rare earth oxides (REOs). The Chlorination operates according to a principle similar to oxidation. The most frequently utilized chlorinating chemicals involve NH_4_Cl, FeCl_2_, and MgCl_2_ [81]. Using NH_4_Cl-based chlorination, Itoh et al. [82] explored an effective procedure for recovering and reusing metallic iron and rare earth elements from NdFeB magnet residue. The primary Nd_2_Fe_14_B phases, Fe-B, and α-Fe, through a 3 h heat treatment process at 300 °C, were transformed to NdCl_3_ and obtained a 90% yield. The researchers Lorenz and Bertau [83] used a solid-state chlorination method to produce gaseous HCl by decomposing NH_4_Cl at high temperatures. The magnetic metal parts were dispersed into an acetic acid buffer solution and interacted with hydrochloric acid (HCl) by producing the metal chlorides and REEs of 84.1%.

Similarly, Uda et al. [84] used the deoxidizing agent molten Fe alternatives of activated carbon (AC) and utilized FeCl_2_ to selectively chlorinate REEs from magnetic waste. The REEs were separated as chlorides using vacuum distillation at 1000 °C in an Ar atmosphere, causing the combination of DyCl_3_ and NdCl_3_ purely with a 76% yield capacity. This process is eco-friendly and does not release any hazardous chemicals or fluids, while the extracted REOs can be utilized as ingredients for making electrolytic oxides. Furthermore, the study carried out by Shirayama et al. [81] by using MgCl_2_ for the selective chlorination and recycling of REEs from magnetic alloys resulted in the separation of Nd and Dy in molten MgCl_2_ with 80% yield extraction. The two important extractive pyrometallurgical techniques used to treat e-waste are roasting and smelting, which involve heating the material at temperatures above 1000 °C, usually without any addition of water. Similarly, to other heat treatments, roasting is exothermic and involves pre-treatment. In the carbothermic reduction method, a reducing agent (such as carbon or coke) is heated together with the cathode material, resulting in the formation of carbonaceous waste (e.g., charcoal and graphitic carbon) and a mixture of metal alloys along with impurity metal oxides. Ultimately, the mixture is purified to extract highly pure or precious metals. The study of Liu and his team [85] invented a hydro-carbonation hydrolysis technique to extract the REEs from de-magnetized NdFeB PMs waste having a particle size of 150 µm > by applying the biochar of particle size < 150 µm, which was produced from the pyrolysis of recycled sawdust as the extracting agent. In addition, they applied carbothermal reduction to roasted NMC cathode material at 650 °C for 30 min with 10% carbon. This technique efficiently recovered 98.68% of Ni, 98.08% of Mn, and 93.33% of Co.

Pyrometallurgical operations use high temperatures to recover REEs, although they often occur in a variety of alloys or mixed compounds that require purification using hydrometallurgical treatment. There has been a lot of interest in the latest research on REE recovery from NdFeB magnets [86]. Stopic et al. [87] described pyrometallurgical REE from NdFeB magnets by involving the oxidation of the material in the presence of oxygen in a muffle furnace at 1000 °C for 2 h. Then, the reduction in the magnet through a carbothermal procedure to split it into a metallic substance and an oxide phase, which is rich in RE_2_O_3_, is performed. The oxidation compounds’ qualitative analysis showed chemical compositions as follows: as Fe_2_O_3_, NdFeO_3_, Fe_3_O_4_, Pure Fe, Dy_2_O_3_, Pr_2_O_3_, and Nd_2_O_3_ with their respective values of 53.41, 16.45, 10.37, 5.22, 1.28, 1.07, and 0.45 wt.%. The separation of NdFeB magnets into RE_2_O_3_-enriched slag phases and metals through pyrometallurgical smelting methods by producing REEs. The oxidizing magnet materials were heated during the reduction smelting process at 1500 °C, 1400 °C, and 1350 °C in an inductive heat furnace. According to the research findings, the extraction efficiency of REEs improved at higher melting temperatures. The resulting metal possessed the most Fe (92.3% by weight) when it was smelted at 1500 °C, whereas the slag had the REEs (47.47%) yield. In the metallic state phase, Nd, Pr, and Dy were separated with 98.1%, 98.9%, and 97.5% yields.

The REEs extraction via molten salt electrolysis, a thermal-electrochemical technique, has been thoroughly investigated. The selective dissolution of Nd and Pr from NdFeB PMs into molten LiF-CaF_2_ material as RE^3+^ ions, studied by Yang et al. [88]. The electrolysis approach converts the RE^3+^ species to RE metals, while the remaining metals remain as Fe and Fe_2_B phases. In addition, Jeon et al. [89] developed a molten electrolysis-induced extraction approach by using the LiCl-CdCl_2_ melted materials at 500 °C, for the extraction of RE metals from PM waste.

The majority of Nd in NdFeB magnets was recovered using CdCl_2_, and the remaining Fe-B phase was filtered out. The recovery of Nd was achieved via liquid cadmium (Cd) cathodic electrolysis in a LiCl, KCl, NdCl_3_, and CdCl_2_ framework, resulting in a Cd-Nd alloy. The ingot was subsequently distilled under vacuum, which removed the Cd while keeping the solid, highly pure Nd. The purified Cd may be reclaimed and used again in subsequent electrolysis procedures. The present technology makes molten salt electrolysis costly in terms of energy, whereas this approach has reduced gas emissions. Initially, the NdFeB magnets reacted with biochar to synthesize an NdFeB-CH alloy. The alloy, which is produced, was then milled into a fine particle size and soaked in water to create RE-Hydroxides (REOHs), which enable the segregation of Fe-based metals via magnetic separation. Ultimately, the REOHs underwent calcination to produce RE_2_O_3_ as the resulting product. The yield of REEs achieved 88.4% under optimum working conditions, with the REOHs obtaining a purity of 99.43%.

### 9.3. Glass Slag Method (GSM)

GSM has been extensively used to recover Nd from spent NdFeB alloys. This technique involves the chemical interaction between Nd, Fe, and B in alloys and glass slag materials such as CaO and SiO_2_. A complicated molten system is formed when wasted magnets and flux are melted in a high-pressure environment of nitrogen or oxygen and temperatures of 1300–1500 °C. The REEs can be selectively oxidized and dissolved into the slag phase by precisely manipulating the temperature and ambient conditions. So, only Nd is removed, leaving the remaining slag with other elements. Saito et al. [90] produced an Fe-B alloy from spent NdFeB alloys via molten boron trioxide (B_2_O_3_) slag contact, extracting the Nd. Likewise, in Bian et al. [91] study, they recover the REEs from scraped NdFeB PMs by using diverse FeO-B_2_O_3_ flux systems. Although the use of a FeO-B_2_O_3_ extractant at 1300 °C yielded REOs with 98.4% purity and above 99.5% extraction yield. Moreover, Abrahami et al. [48] carried out the low-temperature leaching of H_2_SO_4_ and high-temperature slag separation by applying CaO, Al_2_O_3_, SiO_2,_ and CaO-CaF_2_ slag strategies for the extraction of RE with an efficiency of 99%.

## 10. Hydrometallurgy Method for Recovering REEs

The hydrometallurgy technique is significantly utilized for metal recovery by applying aqueous solutions to extract REEs from both primary and secondary waste products. There are two steps involved: initially, the leaching, which is dissolving electronic waste components in a chemical aqueous solution, and then recycling, which is selectively removing the dissolved metals from the leachate. This method of recycling involves the dissolution of REEs individually and then precipitating them as oxalates, fluorides, or double sulfates. The process begins with the leaching of magnetic waste, followed by efforts to isolate certain REEs from NdFeB species, such as Nd, Dy, and Pr, using ionic solvents, solvent extraction, and ion exchange methods [92]. The solubility of metals in water and acids is the primary solvent. So, a suitable chemical solution is applied to the waste product or residue during the leaching phase to facilitate the extraction of the target metals [93]. By utilizing this approach initially, it predominantly concentrated on comparatively pure magnet materials and used industrial byproducts, rather than on EOL magnetic materials, which frequently contain a mixture of various useless ingredients. Previously, the Sm-Co magnets were recycled due to their better economic worth and simpler composition than NdFeB PMs, which include cheaper Fe around 72 wt.%.

In addition, this process offers superior recovery yields and selectivity, demonstrating its exceptional utility for treating complex waste materials. Nevertheless, the process is challenging because NdFeB magnets have protective layers like Ni and different REEs species such as Nd, Dy, Pr, and Tb. To recover the REEs via hydrometallurgy from EOLs-PMs, particularly from weak waste streams, it is necessary to selectively dissolve, concentrate, and separate REEs from the dominating base metals, while confirming overall metal extraction. So, the extraction of REEs from low-concentration streams requires improved separation strategies involving innovative approaches as mentioned above. Thus, with these challenges, hydrometallurgy is still an important and useful technique that acquires REEs from so many different sources.

The leaching liquid is mixed with an organic solvent that contains REEs’ affinity as an extracting agent. To facilitate the transit from the aqueous to the organic form, this interaction allows for the successful separation of REEs. The efficient separation of target REEs from leach solution metallic compounds requires extractive agents that are chosen based on their selective affinity characteristics. The succession of two extracting agents’ examples is di-2-ethylhexyl phosphoric acid (D-2EHPA) and tri-n-butyl phosphate (T-nBP) [94]. The solvent extraction procedure, which is eco-friendlier using ionic liquids, has recently been the subject of research [95]. In ionic liquids, no air pressure is involved, and they are naturally electrically conductive and also chemically stable. Likewise, in comparison to traditional organic solvents, it is more secure and sustainable due to its non-volatile properties. Despite ongoing research, ionic liquids can efficiently extract REEs even at high iron quantities, which could be used to recycle magnetic waste products [96].

Additionally, waste product recycling can be an important part of reducing pollution in the surroundings. The recycling initiatives were driven more by concern for the environment than by financial gain until NdFeB PMs became widely used in the 2010s. Unfortunately, hydrometallurgical processing has a few drawbacks that restrict it from being widely utilized. By including long process chains, a lot of reagents, expensive treatment costs, and complicated equipment. Similarly, this method is better than pyrometallurgy despite hazards such as waste products, heavy metal pollution, and harmful effects [92]. Moreover, it also delivers safer and simpler-to-handle working conditions, better recovery efficiencies, and no slag formation. The fact of choice by using this method is that it uses less energy, produces fewer harmful gases and particles, and lowers emissions of greenhouse gases (GHG) [97].

### 10.1. Pre-Treatment Process

In hydrometallurgical recycling processes, the NdFeB needs a pre-treatment process initially that might impact the leaching efficiencies, selectivity, and overall metal recovery. The spent magnets usually contain the coatings (e.g., Ni/Cu, epoxy), corrosive, binder products, and complex multi-phase micro-structures composed of NdFeB grains and Nd-rich intergranular phases. So, such complications with NdFeB waste hinder the direct chemical dissolution and can lead to excessive acid usage and poor separation if not properly addressed. Thus, the pre-treatment strategies are compulsorily applied to the physical and chemical conditions of the magnetic waste before leaching. These procedures typically involve the demagnetization, mechanical size reduction (crushing, milling), and the removal of surface coatings and other contaminations.

#### 10.1.1. Oxidizing Roasting

The REEs recycling from magnetic waste, whether it be from industry or electronics at the EOL, requires a pre-treatment oxidative roasting method. The research by Yoon et al. [98] oxidatively roasted sintered magnet waste at 500 °C and bonded magnet material at 700 °C. By following, the magnetic waste was leached with 2 mol/L^2^ of H_2_SO_4_ at 50 °C for 2 h, and then performed the double salt precipitation. In comparison to untreated magnets, Nd_2_O_3_ and Fe_2_O_3_ dissolved much more quickly; however, Nd_2_O_3_ could not be leached selectively over Fe_2_O_3_. Similarly, to Layman and Palmer [99], Nd recovery yielded 99.9% and Fe 95%. Alternatively, the comprehensive decomposition of the magnets might be achieved through the usage of HCl [100]. The REEs contained in the produced leachate are subsequently recovered via precipitation using oxalic acid (OA) or hydrogen fluoride (HF), which causes the formation of the respective oxides or fluorides.

#### 10.1.2. Demagnetization

Demagnetization is a vital step in the initial processing of hydrometallurgical recovery of NdFeB-based magnet materials. Two viable methods can be used to accomplish demagnetization. Initially, the use of thermal demagnetization involves NdFeB PMs that undergo an irreversible decay of magnetism when heated beyond certain thresholds. The loss grows worse as the temperature rises until the material completely loses its magnetic properties at or above its Curie temperature (T_c_). Consequently, heat demagnetization has been studied by Ozdemir, A. U.’s research group [101] more extensively as a superior method. The impact of heating at various temperatures, like 300 °C, 350 °C, 400 °C, and 650 °C, was analyzed, given that temperature might affect the chemical and physical characteristics of the recyclable product. In NdFeB magnets, the T_c_ typically varies between 320 °C and 350 °C. This corresponds to the temperature at which the magnetization completely diminishes. Similarly, in the second method, the magnet is subjected to a decreasing cyclic magnetic field, which causes the material’s magnet domains to become randomly distributed and gradually vanish. After such procedures, the permanent magnetism can lead to powder agglomeration. Also, ineffective demagnetization causes the procedure to be unsuitable for recycling purposes. Thus, demagnetization saturation is very important to facilitate the steady input and compaction of powder throughout magnet manufacturing.

#### 10.1.3. Grinding and Size Reduction

The effective recycling of NdFeB PMs critically depends on an effective pre-treatment process, in which the mechanical grinding and crushing play a vital role. The grinding approach used for the reduction in the particle size of bulk NdFeB magnets disrupts the metallic matrix and also improves the exposed surface area, leading to the subsequent H_2_ decrepitation, selectivity, and leaching of RE-rich phases. In addition, the proper control of milling parameters is important to balance the particle size reduction, oxidation prevention, and phase integrity, which directly impact the yield of the recycling. For example, the study carried out by Pawel Friebe and his teams, in the grinding process, a specific amount of crushed NdFeB PMs was added to a container along with the ethanol and tungsten carbide milling balls. The mass ratio of the grinding balls to the sample material remained at 10:1. Whereas, the milling procedure was carried out utilizing a rotating miller performing at a rotational velocity of 300 rpm. Additionally, an experiment was carried out for milling times (without break) of 0.5, 1, 5, 10, and 15 h continuously. The analysis of the distribution of particle sizes was obtained after each particular grinding period. After grinding, the bowl was sieved to remove the grinding balls and milled powder. The ethanol to be removed, the powder solution in ethanol was placed in a container and heated in an oven in the laboratory at 40 °C for 12 h.

### 10.2. Leaching Process

#### 10.2.1. Selective Leaching Process

Leaching methods offer a flexible way to dissolve magnets and magnet material. The selective leaching reduces acid usage, operational expenses, and extraction productivity compared to complete leaching. To selectively leach Nd, melt the magnet in the presence of oxygen at 700 °C, and then leach with 4 mol/L of H_2_SO_4_ for just 3 h at a temperature of 70 °C by controlling the pH values [47]. By using this approach, Fe predominantly transforms into Fe_2_O_3_, whereas Nd oxidizes to produce acid-soluble Nd_2_O_3_ and NdFeO_3_. Therefore, this method could only recover around 70% of the Nd REEs. In addition, after the oxidative roasting at 900 °C for 6 h has been performed to selectively leach the sequential dispersion of 0.02 mol/L of HCL for 2 h, causing a 99% extraction yield of REEs and lower than 5% mixed dissolution of Fe [102]. The magnetic alloy and waste materials have significant Fe content, making selective leaching of REEs from magnet waste more difficult. Although the heat treatment improves the REEs extraction selectivity between Fe-metal and other various transition metals. The rate constants for leaching of the respective oxides have been investigated and correspond to the sequence Nd_2_O_3_ > NdFeO_3_ > Fe_2_O_3_ [103].

The roasting of NdFeB sludge differs substantially from EOL magnet treatment because it generates unique structural changes, which are an important factor when assessing the way REEs leach out. The roasting process has three stages: tight layer structure temperature ranges ≤400 °C, loose layer structure between 400 and 500 °C temperature range, and non-soluble REEs structures of ≥600 °C temperature range. Initially, the Nd leaching productivity is low due to a dense Fe_2_O_3_ layer that hinders dissolution. However, a more porous oxide layer is produced as the treatment temperature is increased, which improves the extraction of REEs. The DTA-TGA characterization was used to analyze the oxide formation at different temperatures, such as Nd_2_O_3_, NdFeO_3_, and Fe_2_B at 688, 686, and 902 °C. Therefore, the higher temperatures (600 °C) lead to NdFeO_3_ phase development and particle agglomeration, by decreasing REE leaching performance [104]. Subsequently, the atomic-level structural differences, different oxides exhibit diverse leaching behaviors. Thus, to effectively recover REEs from wasted NdFeB PMs, they should be calcined at 900 °C and dissolved with HNO_3_ or HCl [105]. The simplicity of recovery at the final stage of the recycling process has led to concentrated salt solutions being studied as ecologically useful leaching agents. The researcher Ding et al. [106] investigated the practically complete recovery of Nd by utilizing ZnCl_2_ as the leaching reagent, obtaining an extraction value for Nd and Fe beyond 1 × 10^5^. Therefore, the current experimental research shows that the [ZnCl_4_(H_2_O)_2_]^2−^ mixture in ZnCl_2_ solution acts as a Bronsted acid, improving the removal of Nd_2_O_3_ phase. Therefore, pre-treatment through heating is not a single approach for the selective dissolving of REEs; chemical leaching methods have also been explored in many previous studies.

#### 10.2.2. Complete Leaching Process

The complete leaching method assures the complete dissolution of all species within the NdFeB PMs waste, thereby facilitating their subsequent isolation via selective methodologies predicated on separated chemical and physicochemical characteristics. This process uses an extremely acidic atmosphere with a high enough acid concentration to dissolve the magnet completely. The inorganic acids, such as Hydrogen Sulfate (H_2_SO_4_), Hydrochloric (HCl), and Hydrogen Nitrate (HNO_3_)_,_ are extensively acknowledged according to Abdulvaliyev, R et al. [107] as highly effective leaching agents for the complete extraction of metals, predominantly owing to their distinct acidity and cost-effectiveness. Among these, HCl and HNO_3_ exhibit enhanced efficiency relative to H_2_SO_4_, presumably due to the adsorption of chloride and nitrate ions on the metallic surface. Consequently, the effectiveness of the dissolution process is notably affected by various parameters, such as the type of acid, temperature, solids-to-liquids ratio, acid concentration, and reaction time. The two researchers, Layman and Palmer [108], proposed a room-temperature leaching technique employing H_2_SO_4_ for clean industrial scrap, with or without roasting. Subsequently, the Nd can be re-formed as a salt of two sulfates, which is easily transformed into NdF and NdO. This replacement of surface (OH) groups promotes the removal of REEs, which increases the overall performance of extraction [109]. The complete dissolution of NdFeB magnetic waste under such circumstances is achieved.

As a leaching agent, HCl is usually preferred over HNO_3_ due to environmental and economic considerations. The efficient element extraction from NdFeB waste can be achieved by using a complementary reagent mixture of inorganic acids and chelating agents. By applying the optimum conditions, such as 50 g/L of tartaric acid (C_4_H_6_O_6_) with 6 mol/L of HCL at 40 °C, the recovery efficiency for REEs and Fe was 99.2% and 67.9%. After the extraction of the majority of metals, it is also imperative to start the separation of impurity metals. The Fe^2+^ ions contained within the effluent may be oxidized to Fe^3+^ by the addition of a 10% hydrogen peroxide (H_2_O_2_) solution. Also, by adding oxalic acid, RE oxalates can be selectively precipitated, and the leaching acid can be recovered and recycled. The composite that contains Nd, Pr, Co, Al, and Fe can be produced by adding 15% NH_4_OH and then filtering the mixture [110]. There are significant health and environmental risks associated with the production of hazardous acidic gases (such as Cl_2_, SO_3_, and NO_3_) by conventional inorganic acids during leaching. On the other hand, organic acids have recently come to light as potential safer and less harmful replacements.

In addition, experimental research has demonstrated that organic acids’ intrinsic chemical characteristics dominate in determining their leaching effectiveness. Similarly, the study by Gregoric M et al. and their team [111] scientifically investigated the recovery of REEs through leaching with different organic acids. In this research, they studied the NdFeB magnets through a citric acid (C_6_H_8_O_7_) solution with a concentration of 1 mol/L, which leached nearly all of the Nd after 24 h. The REEs retained solubility as citrate complexes at pH values between 2 and 5, but higher pH values caused RE citrates to precipitate, and it was concluded that the pH value of the solution is very important. Under ideal circumstances, studies comparing malic, glycolic, and ascorbic acids showed that the initial two performed better than the ascorbic acids, with extraction efficiencies for Nd, Dy, and Fe reaching over 95%. Moreover, enhanced performance is probably linked to the higher density of hydroxyl (OH) and carboxyl (COOH) groups, which make it possible to better coordinate physically [112]. The latest literature has added clarity to the criteria for choosing appropriate organic acids for leaching procedures. The research performed by Belfqueh et al. [113] on the leaching performance of NdFeB magnets by investigating numerous organic acids, such as tartaric acid (C_4_H_6_O_6_), acetic acid (C_2_H_4_O_2_), formic acid (CH_2_O_2_), and citric acid (C_6_H_8_O_7_). Although via the optimization, the Nd, Dy, and Pr leaching yields exceed around 90% at 60 °C by applying the acid concentration between 1.6 and 10 mol/L with a solid-to-liquid ratio of 0.5–5%. In conclusion, the most effective leaching agent was C_2_H_4_O_2_ acid, which had a lower pKa value and was better suited to form a stable group with REEs.

#### 10.2.3. Inorganic Leaching Solvent

An exothermic reaction occurs during the leaching of inorganic chemical acids. The NdFeB PMs dissolution in diluted acid, hydrogen gas interacts with the ions of Nd, Fe, and B present in the solution. Then, the leachate contains the Fe^2+^ or Fe^3+^ ions, depending on the type of acid and leaching conditions. Also, the leachate mostly contains Fe^2+^ ions when HCl and H_2_SO_4_ are used for leaching, and the Fe^3+^ ions are employed via HNO_3_. The study of Figueira et al. [114] utilized four leaching agents, NaOH, HCl, HNO_3_, and H_2_SO_4_, and various pH-adjusted precipitate techniques to extract Nd from NdFeB PMs. The H_2_SO_4_ and HCl exhibited the most effective leaching behavior, achieving a 100% leaching rate and a 95.68% recovery efficiency for Nd. Similarly, Yoon et al. [98] used H_2_SO_4_ at different concentrations between 2.5 and 3.5 mol/L, pulp density of 110.8 g/L, 750 rpm, and temperatures ranging from 30 to 70 °C. The amount of Nd in the e-waste powder was entirely changed to Nd_2_(SO_4_) after 4 h of leaching.

Researcher Laatikainen et al. [115] employed HCl and HNO_3_ as leaching agents to extract Nd, Dy, and B species, with an excessive yield of about 80% into the solution at 5 min intervals and at ambient temperature. The REEs at 80 °C were entirely dispersed within 5 min, achieving a recovery rate of 98% for Nd and 81% for Dy from the magnetic waste, while over 99% of Fe was dissolved into the solution. Although the leachate’s pH reached up to 3, it led to the formation of Fe (OH)_3_, which removed the Fe species. In addition, Itakura et al. [116] used ethanol, ethanedioic acid (H_2_C_2_O_4_), and NaCl as precipitating agents. These findings demonstrated that such precipitants induce the growth of insoluble Nd compounds. Nevertheless, the precipitants H_2_SO_4_/ethanol or H_2_SO_4_/NaCl are utilized where the Fe species are present. The extraction of Nd_2_(C_2_O_4_) from the magnetic waste was performed through an aqueous solution of 3 mol/L HCl and 0.2 mol/L H_2_C_2_O_4_, exhibiting 99.8% purity for 8 h, where the Nd is present around 99%. Likewise, Behera and Parhi [117] employed HNO_3_, by altering in concentration, temperature, and particle size, 800 rpm, 1% pulp density, 106–150 μm particle size, and 180 °C temperature to observe the extraction rate. The amount of Nd and Fe extracted from the waste magnets was 99.99% and 99.9%, respectively.

#### 10.2.4. Organic Leaching

Leaching with organic acids is better for the environment than leaching with inorganic acids. Therefore, the organic molecules of acids like C_2_H_4_O_2_, C_6_H_8_O_7_, C_4_H_6_O_5_, and C_2_H_2_O_4_ are used in the leaching process to recover and remove specific metals. However, compared to inorganic acid leaching, organic acid leaching causes no hazardous emissions, and the resultant fluid waste is biodegradable. To extract and recover REEs, organic acids like C_6_H_8_O_7_ and C_2_H_4_O_2_ are commonly utilized. In comparison to inorganic acids, these two organic acids are cheaper and can effectively disintegrate REEs. Thus, through this procedure, the REEs recovery of Nd, Dy, and Pr, the yield might achieve 95% at 25 °C for 24 h, through the usage of these two C_6_H_8_O_7,_ the C_2_H_4_O_2_ acids. This phenomenon occurs because Fe and Co exhibit equivalent solubility quantities as REEs in both acids [118]. The C_2_H_4_O_2_ acid, having a high REEs recovery rate, is also capable of dissolving other elements like Co and Ni that are present in magnetic waste. The usage of C_6_H_8_O_7_ and C_2_H_4_O_2_ for the extraction of magnetic waste mainly depends on the acid concentration. There are also other acid usage criteria, including leaching duration, pulp density, and amount, as well as concentration. The extraction of Nd, Dy, and Pr was consistently measured between 95 and 96% over a range of (solid-to-liquid) S:L ratios [111]. Therefore, the researcher described the impact of S:L ratios (1:20), which shows the best ratio for the REEs (Nd) recovery is via the use of C_6_H_8_O_7_ acid, resulting in the recovery rate of 72.8%. Thus, the REEs extraction rate decreased at an S:L ratio (1:50) to 59% [119]. Such variations in the S:L ratios displayed a negligible impact on C_6_H_8_O_7_ usage. The other various organic acids, such as (formic) CH_2_O_2_, C_6_H_8_O_7_, (tartaric) C_4_H_6_O_5_, and C_2_H_4_O_2_, can be utilized in magnetic waste leaching. However, both CH_2_O_2_ and C_4_H_6_O_5_ acids are considered less productive for REEs recovery, with recovery efficiencies of only 75% and 35%, respectively. This result demonstrated that the interaction occurs between the NdFeB magnet and CH_2_O_2_ acid, causing the formation of the Nd (HCOO)_3_ compound that has lower solubility. Also, the C_4_H_6_O_5_ acid reacts with the NdFeB magnet and forms Nd (OH)_3,_ which is also insoluble [113].

Consequently, such results show a lot of potential for both organic and inorganic acids as a leaching agent for REEs. As a result, the above-mentioned results for metal leaching make the leaching procedure more demanding for organic acid consumption throughout both the leaching and Fe removal phases, while generating significant waste streams and increasing operational expenses. The other traditional approaches for Fe extraction involve raising the pH to 3 to precipitate Fe (OH)_3_. Nonetheless, this method adds complications to the process and further reduces the recovery rate because of the adsorptive capturing of REEs by Fe (OH)_3_ colloidal.

#### 10.2.5. Mechanochemical Leaching

A hybrid extraction method called mechanochemical leaching is employed through initially physically activating metallic elements and then the dissolution of it by using specific chemicals. The mechanical activation of REEs-based materials decreases their thermodynamic stability and particle size while improving REEs’ leaching selectivity, kinetics, surface area, and crystallinity [120]. The high-energy ball milling method is commonly employed for the mechanical activation of NdFeB magnetic waste. In addition, the primary operational parameters, such as chemical activators, ball-to-powder ratio, milling time, and speed, need to be optimized to promote the mechanochemical reaction mechanism [119]. The research on NdFeB magnetic waste via leaching efficiency can be improved after being ball-milled for 1 h at a rotational speed of 300–500 rpm. Therefore, the mechanical activation of NdFeB wastes subject to such conditions facilitates the REEs extraction efficiency around 96–99%, and significantly improves the rate of recovery through traditional inorganic acid leaching techniques. Likewise, activator compounds are also added to the milling process to initiate the in situ mechanochemical process and further enhance productivity. More water-soluble byproducts are produced when those activators interact with REEs. Thus, it implies that only small amounts of weak acids or even water are needed for dissolution, which makes the process more environmentally friendly. Similarly, the mechanochemical process of NdFeB waste utilizes Fe_2_(SO_4_)_3_ as an activator, where the activator to magnet waste mass ratio becomes an important parameter. The co-milling of Fe_2_(SO_4_)_3_ to waste magnet of ratio 4:1 has demonstrated the capability to attain REEs recovery yields of up to 97% through subsequent aqueous extraction conducted at room temperature (25 °C) over a period of 10 to 25 h. These results show that mechanochemically aided leaching could be a great way to recover REEs from NdFeB magnets that are no longer in use, and it would be good for the environment [121].

## 11. Separation and Purification of REEs

There have been numerous methods for the separation and purification of REEs from dissolving solvents. However, the most popular methods include chemical precipitation, solvent extraction, selective crystallization, and ionic liquid extraction [122].

### 11.1. Chemical Precipitation

In the Nd-H_2_O and Fe-H_2_O system, the RE^+3^ ions are more stable up to a pH value of 6, whereas the Fe^+3^ ions precipitate at a pH value of 2. Therefore, by reducing Fe^+2^ to Fe^+3^ followed by changing the pH, Fe^+3^ was eliminated selectively [123]. The selective Fe precipitation approach requires adding alkaline reagents due to the persistent presence of Fe^+2^ and Fe^+3^ in the solution, as considered one of the drawbacks of the method. Although the precipitation of reactive elements can also be achieved through the use of metals and Na^+^, K^+^, and (NH_4_)^+^ ions. The solar energy industry’s wastewater was treated with ultrafine NdFeB wastes to recover fluorine, as reported by Sun Chi et al. [124]. Subsequently, this method allows the precipitation of Nd(OH)_2_F, which recovers the REEs and reduces the concentration of fluorine ions in wastewater. Additionally, the recovery process requires the combination of KF and HNO_3_ ions within an aqueous solution, maintaining a pH value of 0. However, this approach is not yet suitable and cannot provide a complete solution to the detrimental effects of F-emission in the environment. Thus, the REEs produced from the leached fluids of NdFeB wastes can be selectively precipitated through the usage of organic oxalic acid (H_2_C_2_O_4_) [125]. In addition, the selective precipitation of REEs and Fe is advantageous for the other complicated salts precipitation due to the minimization of Fe^+2^ and Fe^+3^ interferences [126]. The leachate modification can be performed by adding M_2_SO_4_ (M for Na, K, or NH_4_ ions) to produce [RE, M] (SO_4_)_2_. nH_2_O precipitates in the mixture. However, a detailed procedure will be required, including the reprecipitation of H_2_C_2_O_4_, redissolution of HCl, and the calcination process, to produce the final pure REEs products [127,128].

### 11.2. Solvent Extraction

The REEs can be precisely separated from active leaching solutions through a solvent extraction process, which allows the regeneration and reuse of the loaded organic phase following the stripping process. Elust et al. [129] used HCl to specifically leach Nd and Dy from the subjected NdFeB heated sample. As a result, a large amount of Fe was also removed through hydrolysis, which released Fe (OH)_3_. Furthermore, the extractant Di-2-ethylhexyl Phosphoric acid (D_2_EHPA) was used to remove the Nd and Dy from the leachate in two phases. Whereas the usage of HCl stripping nearly 95% of Nd and Dy was extracted from the loaded organic phase, while also removing a small amount of Fe.

### 11.3. Selective Crystallization

The selective crystallization of REEs and Fe separation is facilitated based on their physical properties and following the elimination of the requirement for chemical reagents. The study of Ciro et al. [130] used the inverse solubility of RE sulfates to break up demagnetized NdFeB waste into pieces smaller than 420 μm. Likewise, the magnetic powder was completely leached out by using (NH_4_)_2_S_2_O_8_. Additionally, the Nd-sulfate crystals were also extracted at a pH value of 0.1 and a temperature of 75 °C, yielding 96% purity of crystals. Since most of the RE ingredients contain a combination of RE compounds, which have been the main focus of developing methods to effectively separate the REEs and Fe. However, the difficulties in the separation of REEs were faced due to their similar chemical properties [131]. Furthermore, the solvent extraction strategies usually consist of REEs separation, which is well established in terms of vibrant extraction and separation steps [132]. Additionally, the main focus of the latest research is the extraction of precious metals with limited attention to the small amount of almost 1% B. During the NdFeB PMs leaching procedure, the B ions, like B^+3^ or H_3_BO_3_, are also incorporated into the solution. These strategies have been made to recover and extract the main target materials of REEs once the B is enriched [133].

## 12. The Recycling Methodologies Optimization for NdFeB PMs via Machine Learning (ML)

The recycling of NdFeB waste has extensively attracted attention due to the viability of REEs and growing demands for PMs in sustainability and other advanced technologies. The typical fields of ML application in REEs are related to the detection, the ore classification and exploitation, and the research (processing, properties, composition).

Therefore, it is compulsory to recycle the NdFeB waste and optimize its leaching parameters via ML to achieve maximum recovery with a small amount of chemical utilization. The recycling of REEs from NdFeB waste in industries has been achieved mostly via hydrometallurgical and pyrometallurgical methods [134]. Meanwhile, pyrometallurgical methods typically employ high-temperature processes, such as smelting and alloy separation, which are energy-intensive and may result in partial loss of REEs. In comparison with the hydrometallurgy approach, which relies on aqueous chemistry for the selective dissolution and separation of REEs, this method offers better control over product purity. Likewise, before the main recycling step, several important initial procedures involving crushing, grinding, calcination, and oxidation are employed to enhance process efficiency [113]. Consequently, such a process facilitates the breakdown of the NdFeB matrix, promotes the oxidation of Fe-rich phases, and enhances the selective leaching process. Therefore, companies and industries prefer hydrometallurgical methods to recycle the NdFeB magnetic waste due to the use of less energy and also the production of pure magnetic products. In addition, these processes are more adaptable to process optimization and integration with environmentally sustainable practices, making them attractive for scalability and economically viable recycling of NdFeB magnets. The hydrometallurgical procedure involves the most important part, which is the acid leaching of REEs from NdFeB magnetic wastes [102]. The various factors that affect the yield of REEs leaching during recycling include oxidation calcination parameters (e.g., time, temperature), raw materials characteristics (particle size, composition), acid leaching parameters (pulp density, stirring speeds, acid types and concentration), and their interactions [120,126]. In addition, there is no guarantee for achieving an excellent REEs rate by using optimum parameters from various studies due to the variation in NdFeB magnet compositions, types, and manufacturers. Thus, to achieve the optimal leaching parameters, an enormous amount of effort, time, and chemical constituents must be required to conduct many repeated experiments. Therefore, the current traditional methodologies do not fully reflect the complicated cross-influence procedures of various parameters on REEs leaching.

In order to address the aforementioned challenges in both processes, Machine Learning (ML) has the ability and has been employed to present a significant advancement. In addition, the ML can analyze huge datasets and can identify their relationships and patterns [135]. Moreover, this approach is superior to conventional analytical methods to deal with non-linear problems [136]. Consequently, this capability was exploited and used to establish predictive models, experimental design, shape, and enhance recovery process optimization [137]. In recent years, ML has become one of the most expanding applications for environmental contexts, which includes the prediction of metal and organic pollutant adsorption capacity on biochar, and is beneficial for advancing waste recycling methods [138]. The additional aspects that impact the recycling process are implemented through the interceptive approach in ML. Additionally, the improved comprehension facilitated the more precise control over the recycling process, enhanced the metal recovery yields, and reduced the energy and environmental pollutants [139]. The investigation using ML to combine calcination with acid leaching for the recovery of REEs from spent NdFeB magnets has not been addressed despite all such improvements. The main goal of the ML implementation was to identify the best conditions for metal recovery via water leaching and chlorination of roasted NdFeB magnetic scrap by developing ML-based predictive modeling. There are several surrogate models, some of them remarked in Table 4.

ML is a powerful instrument for analyzing geological datasets. The analysis of these data, usually obtained from the analysis after exploration, has high complexity. Thus, ML methods are also applied to predict REE content in sediments using geological datasets [142].

Regarding the ML application to recovery, the ML-based techniques were employed to optimize the recovery of REEs from NdFeB magnetic waste via calcination pre-treatments followed by acid leaching, and tailoring the complicated multivariant interactions governed by REEs leaching. The leaching efficiency based on particle size, waste composition, and calcination parameters has been analyzed, and ML data set points about 9650 were created for statistical analysis optimizations to the recovery of REEs such as Nd, Dy, Pr, Fe, and Co as an output. Additionally, a total of eight models were compared and used four ML algorithms, where the XGB algorithms showed the highest precision validation, and 5-fold cross-validation sets, as described in Table 5. The optimum conditions obtained for the metal’s extractions were 60 min duration for leaching, a temperature of 95 °C, and a solid to liquid ratio of 125 g/L. Consequently, to mitigate the limitations of recycling methodologies, the ML-based models were implemented to facilitate the intelligent leaching of REEs from NdFeB waste and also elucidate the intricate procedures that affect multiple parameters on REEs leaching. To forecast the leaching efficiency of three main REEs, such as Dy, Nd, and Pr, in addition to Co and Fe, the models considered a total of 24 input parameters which includes the oxidation calcination parameters (e.g., time, temperature), raw materials characteristics (particle size, composition), acid leaching parameters (pulp density, stirring speeds, acid types and concentration). For accurate and precise predictions, the best models were implemented with four algorithms to create a total of 24 different models. Subsequently, to examine the interactions and inducing mechanisms among procedures and the ideal intervals for each parameter, it utilized Partial Dependence Plot (PDP), and Shapley Additive Explanations (SHAP) in conjunction with the best model algorithms such as XGB, RF, SVM, and AdaBoost. Additionally, they incorporated the best models into a sensitive Graphical User Interface (GUI). This GUI has been engineered to expedite the process for researchers and industries seeking to identify the optimum parameters for the leaching of metal recovery from NdFeB magnetic wastes, thereby avoiding the necessity for repeated experimental optimization. Similarly, the NdFeB waste characterization was performed using 16 inputs (such as Nd, Dy, Pr, Fe, B, Ni, Ce, Gd, Co, Cu, Al, Ca, Si, O, and P) that encompassed particle size and elemental compositions [138]. The main result is that the difference in the average REES percentage between the predictive and experimental efficiencies is lower than 5%—REEs efficiency.

## 13. The Ecological Importance of the ML in the NdFeB Recycling Technologies

**Figure 6:**
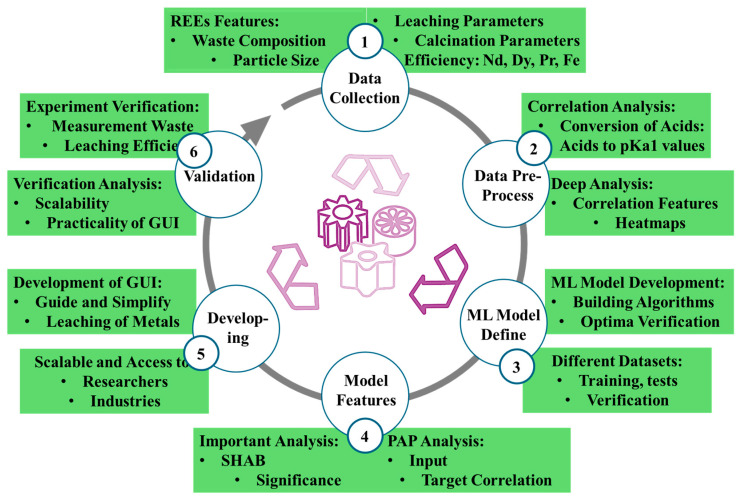
ML-assisted workflow for the recycling of NdFeB magnetic wastes.

The conventional optimization methods and their production of substantial quantities of waste containing hazardous chemicals and metals, which should be disposed of after every cyclic repetition, mainly depend upon trial and error. These hazardous contaminants in the soil and groundwater waste liquids pose serious risks to human health and ecosystems [143]. Additionally, the NdFeB magnets’ e-waste is vital for both resource efficiency and environmental protection through their utilization and re-use of REEs from their secondary sources. Likewise, the NdFeB waste through the pyrometallurgical pre-treatment and acid extraction process has demonstrated its effectiveness for the recovery of REEs [144]. However, the substantial optimization trials are necessary due to the intrinsic diversity in the NdFeB magnetic waste composition to identify the best process parameters. Consequently, these results show a significant use of labor and chemicals, which increases environmental concerns enormously [100]. The ML-based algorithms for the recycling process of NdFeB magnetic waste have been employed to optimize the process parameters such as particle size, acid and calcination parameters, as well as the parameters for the waste composition. Additionally, the reduction in the usage of chemicals, human labor, and environmental hazards while avoiding the drawbacks of conventional optimization studies. The innovative GUI allows the direct optimization of process parameters, which significantly reduces the need for extensive testing and the large amounts of associated hazardous waste liquids. Moreover, the GUI capability allows for accurate and effective resource utilization to quickly estimate leaching rates by using experimental particle size measurements and waste composition. The additional reductions in environmental effects are achieved by employing only the required quantities of energy and chemicals, made possible by this degree of accuracy in process control. The additional advantages include reducing pressure on main mining operations and bolstering the circular usage of essential metals through the promotion of REE recovery from NdFeB waste products. Similarly, the substantial environmental degradation, such as soil degradation, deforestation, and water contamination, is often associated with the REEs mining. Consequently, such methods reduce the requirement for primary ores and related ecological damage by recovering the REEs from waste streams [145,146]. Currently, the recovery of REEs from NdFeB waste utilizing the efficient ML-based methods is a major step forward towards achieving environmental sustainability. It is necessary to update databases and machine learning algorithms [147]. Some efforts have been made, for example, in the optimization of the coercivity [148]. Figure 6 shows a detailed schematic analysis of the main factors involved in ML-based models for the recovery of REEs from NdFeB magnetic waste.

Thus, in an effort to reduce their environmental impact without losing profitability, it offers a glimmer of hope by maximizing resource use, decreasing waste, and encouraging the use of circular metals. Therefore, the effort for this new approach for resources’ protection and the recovery of REEs in the future is to promote sustainable development and environmental protection.

## 14. Conclusions and Future Perspectives

The NdFeB PMs waste management and recycling emerged as a prominent challenge in the modern world, as they are produced during dismantling and manufacturing processes in industries. The main objectives of recycling technologies are the extensive usage of recovered REEs for different applications, such as wind turbines, EV motors, medical appliances, and new upcoming technologies.

The effective recycling of NdFeB waste magnets can reduce the critical materials supply chain from RE-rich countries, energy consumption, implement closed-loop procedures, and also reduce environmental hazards. This review meticulously analyzes contemporary improvements in short-loop, metallurgical, and ML-based recycling techniques exclusively tailored for the NdFeB magnetic waste. The anisotropic NdFeB PMs are produced directly via the short-loop recycling approach, which is economically viable and environmentally friendly. Likewise, the NdFeB waste during the refining process exhibits high concentrations of oxygen, and the incorporation of metallic additives was added to prevent the REEs degradation. Consequently, the volumetric expansion of recycled NdFeB PMs due to the repetitive regeneration strategy caused a reduction in their magnetization saturation and remanence values. Additionally, the direct HD methods have immense challenges for the removal of cross-linked epoxy coating networks due to their intrinsic chemical robustness and impermeability, which provides an outstanding aging resistance. To attain sustainability, it is also essential to reduce the content of carbon and oxygen sufficiently in recycled magnets by considering their potential for reuse in different applications. Moreover, perform this, it is also necessary to employ multiple methods effectively, such as solvent decomposition and the pyrolysis approach. Furthermore, the pyrometallurgical recycling methods have also been extensively investigated, which have potential for large-scale industrial applications, high operational efficiency, and reduced pollutant discharge, making it more ecologically friendly. However, this technique requires considerable energy demands, advanced apparatus, and presents some limitations in achieving precise extraction and separations of REEs. The hydrometallurgical approach provides superior procedure regulations compared to other methods and delivers high-purity REE products with limited solid waste production. In response to these technological limitations associated with the recycling of the NdFeB magnet, there are some other innovative methods, like electrochemical and mechanochemical processes, that have been developed on a laboratory scale to address such limitations. Likewise, these methods have a supercritical extraction rate and an environmentally sustainable approach for the recovery of REEs while exhibiting low carbon development. Nevertheless, there are still some vital challenges related to scalability, operational expenses, and safety issues before they are implemented in industries. Thus, these traditional processes rely on a leaching approach and are highly dependent on chemical reagents, resulting in substantial waste formation and requiring complex multi-stage purification procedures.

Subsequently, to tackle and address the aforementioned limitations, the ML-based models were employed to enable the intelligent leaching of REEs from NdFeB waste. The correlation between the REEs (e.g., Nd, Dy, and Pr), along with other metals and their productivity, temperature, time duration, pulp density, and leaching factors, was investigated thoroughly by using the ML-based model. Likewise, the integration of ML-based methodologies provides a more comprehensive and precise extraction process associated with RE metals. Consequently, the higher yields at reduced expenses have been achieved through the optimization of experimental parameters via the developed ML model. In addition, the extraction mechanisms of RE-Nd metal were extensively investigated and characterized with a key operating variable of temperature. Thus, this significant analysis offers important perspectives for the leaching processes while improving the knowledge of the parameters that influence viability and metal reclamation. To summarize, the incorporation of ML strategies to leach out the selective extraction of REEs from NdFeB waste has become a promising methodology. This pioneering approach provided researchers and industry personnel with robust tools to enhance metal recovery yields and optimize approaches, thereby promoting environmentally friendly usage of limited resources and minimizing environmental consequences.

## Figures and Tables

**Table 1:** The distribution of the weight % of REEs for the RE production in permanent magnets [17].

Element	Nd	Pr	Dy	Tb	Other REEs
wt.%	60–70	0.5–7	0.2–6	0–1	<0.5

**Table 2:** Physical and chemical comparative analysis of all types of magnets.

Magnets	NdFeB	Ferrite	AlNiCo	SmCo
Magnetic Strength	B_r_ = 1–1.4 T BH_max_ = 200–400 KJ/m^3^	B_r_ = 0.2–0.45 T BH_max_ = 30–40 KJ/m^3^	B_r_ = 0.6–1.3 T BH_max_ = 10–88 KJ/m^3^	B_r_ = 0.9–1.2 T BH_max_ = 120–200 KJ/m^3^
Coercivity	Excellent (drops with heat)	High (mostly Sr- ferrite)	Low (Suffer demagnetization)	Very high (Good Thermal Coercivity)
Temperature Stability	Low-Medium (80–200 °C)	Excellent (300–450 °C)	Very good (450–550 °C)	Excellent (300–350 °C)
Corrosion Resistance	Poor (Cu/Ni/epoxy coating required)	Excellent (Oxide ceramic, passive)	Good (Needs Coating)	Very good (Better than NdFeB)
Density	High 7.4–7.6 g/cm^3^	Low 4.8–5.1 g/cm^3^	Medium 7–7.3 g/cm^3^	High 8.2–8.4 g/cm^3^
Synthesis Method	Powder metallurgy	Ceramic route	Casting/Powder metallurgy	Powder metallurgy
Mining Ores	Monazite Ore (2–8% of REEs) and NdPr-rich deposits 26–30% of REEs in ore	REEs free or 0 wt.%	REEs free or 0 wt.%	20–36% of Sm (depends on SmCo_5_ or Sm_2_Co_17_)
Chemical Composition	High (Nd/Pr (25–35%), Fe (60–70%), B (0.8–1.2%)	Very low Fe_2_O_3_ (≈80–90%) BaO or SrO (≈10–20%)	Low-Medium Fe (≈50–60%), Al (8–12%), Ni (15–25%), Co (5–24%)	Very high (Expensive Sm (20–30%) and Co (60–70%)
Mechanical Strength	Brittle: low fracture toughness	Brittle ceramic	Good mechanical toughness	Brittle: Like NdFeB
Typical Cost	High (~25–120 $/kg) (Depends on REEs supply chain)	Very low (3–15 $/kg) (Inexpensive magnet type)	Medium (8–15 $/kg) (depends on Co content)	Very high (150–300 $/kg) (expensive due to Sm and Co)
Common Applications	EV motors, Wind Turbines, HDDs, MRI, and Robotics	Sensors, Motors, and refrigerators magnets	Sensors, High-temp motors, and aerospace.	Aerospace, Army forces devices, and motors

**Table 3:** The distribution share of REEs in the global market for the production of NdFeB and SmCo-based PMs.

REEs Elements	Nd	Pr	Dy	Tb	Sm
wt.%	50–60	20–30	8–15	1–5	21–32.3

**Table 4:** Comparison of several surrogate models [140,141].

Model	Mathematical Expression	Type
Response Surface Model (RSM)	y=Xβ+ϵ *X* = Structure matrix; β = Coefficient matrix	Parametric
Radial Basis Function (RBF)	*y* =$\;\sum_{i=1}^{n}\beta iH\;(||x-xi\;||)$ *H:* RBF function; βi = Coefficient matrix	Parametric
Kriging	*y = q* (*x*)′ β *+ z* (*x*) *q*(*x*): Basics function; β: Coefficient matrix; *z*(*x*): A stochastic process	Semi-parametric
Artificial Neural Networks (ANN)	*y_j_* = f($\sum_{i=1}^{n}.$*w_ji_* *x_i_* − *θ_j_*); Basic artificial neuron model, *w_ji_*; Weightings, *θ_j_*; Neuron’s activation threshold; f = Transfer function	Non-parametric
Support Vector Machines (SVM)	*y = w·Φ* (*x*) *+ b* *Φ*: A function maps the input space to a higher-dimensional feature space, *w* is a weighting vector, and *b* is a Bias term.	Non-parametric
Extreme Learning Machines (ELM)	*y* = $\sum_{i=1}^{K}.\beta$*_i_g* (*w_i_ x_j_* + *b_i_*); *g* = Activation function, *w*: Weighting vector; *b*: Threshold	Non-parametric
Mixed Control Model (MCM)	*MC* = [$\frac{1}{3}\ln\left(1-x\right)+(1-x)$^−1/3^ − 1] *x* = Fractional leaching conversion of the target metal (0 ≤ *x* ≤ 1) *x* = 0: No leaching occurred, and *x* = 1 Complete leaching occurred, $\left(1-x\right)\;$= Unreacted fraction of the solid particle	Non-parametric
Graphical User Interface (GUI) Model	*R*^2^ = 1 − $\frac{\sum_{i=1}^{N}\left(\;{y_{p}^{i\;}-\;y}_{t}^{i}\right)\;.^{2}}{\sum_{i=1}^{N}\left({y_{t}^{i\;}-\;y}^{m\;}\right).^{2}}\;$, *RMSE* = $\sqrt{\frac{\sum_{i=1}^{N}{\left(\;{y_{t}^{i\;}-\;y}_{p}^{i}\right)}^{2}}{N}}$ $y_{p}^{i}$ = Predicted value of metal leaching efficiency, $y_{t}^{i}$ = Actual output value, *y^m^* = Average value of all metal leaching efficiency	Non-parametric

**Table 5:** Comparative results obtained for predictive and experimental efficiencies.

NdFeB Samples	Avg. Predictive REEs Efficiency Values (%)	Avg. Experimental REEs Efficiency Values (%)	Error (%)
S1	81.6	80.6	−1.0
S2	77.0	76.7	−0.3
S3	80.5	75.5	−5.0

## Data Availability

No new data were created or analyzed in this study. Data sharing is not applicable to this article.
